# mTOR inhibition by rapamycin increases ceramide synthesis by promoting transforming growth factor‐β1/Smad signaling in the skin

**DOI:** 10.1002/2211-5463.12039

**Published:** 2016-02-24

**Authors:** Takumi Yamane, Aimi Muramatsu, Sawako Yoshino, Sho Matsui, Mari Shimura, Yoshimasa Tsujii, Ken Iwatsuki, Kazuo Kobayashi‐Hattori, Yuichi Oishi

**Affiliations:** ^1^ Department of Nutritional Science and Food SafetyFaculty of Applied BioscienceTokyo University of AgricultureSetagaya‐kuJapan; ^2^ Department of Nutritional ScienceFaculty of Applied BioscienceTokyo University of AgricultureSetagaya‐kuJapan; ^3^ Department of Applied Biology and ChemistryFaculty of Applied BioscienceTokyo University of AgricultureSetagaya‐kuJapan

**Keywords:** ceramide, high‐fat diet, keratinocyte, mammalian target of rapamycin, serine palmitoyltransferase, transforming growth factor‐β1

## Abstract

Although mammalian target of rapamycin (mTOR) mediates a wide variety of biological functions, little information is available on the effect of mTOR on the functions of skin cells. In this study, we investigated effects of mTOR inhibition by rapamycin on ceramide synthesis in the skin of rats and human keratinocytes and its regulatory mechanisms. The phosphorylation of p70 S6 kinase, which indicates mTOR activation, was induced in the skin of rats fed a high‐fat diet, but this abnormality was reversed by supplementation with rapamycin. Ceramide levels and the mRNA levels of *serine palmitoyltransferase (SPT)* and *transforming growth factor (TGF)‐β1* were suppressed in the skin of rats fed high‐fat diets, but this abnormality was reversed by supplementation with rapamycin. TGF‐β1‐induced *SPT* mRNA expression was blocked by SB525334, an inhibitor of TGF‐β1‐induced Smad2/3 nuclear localization, in human keratinocytes. Rapamycin‐induced *SPT* mRNA expression was blocked by an anti‐TGF‐β1 antibody or SB525334 in human keratinocytes. These results show that mTOR inhibition by rapamycin increases ceramide synthesis by promoting TGF‐β1/Smad signaling in the skin.

**Abbreviations**
AD: atopic dermatitis
HF: high‐fat
mTORC: mammalian target of rapamycin complex
mTOR: mammalian target of rapamycin
PPAR: peroxisome proliferator‐activated receptor
S6K: S6 kinase
SPT: serine palmitoyltransferase
TGF: transforming growth factor
TLC: thin‐layer chromatography

Sphingolipids are essential components of the mammalian permeability barrier, and their presence in the epidermis is critical for barrier maintenance 1. Terminally differentiated keratinocytes, called corneocytes, are embedded in a matrix of extracellular lipid lamellae, consisting mainly of ceramides, free fatty acids, and cholesterol 2. Ceramides, one of the most common sphingolipids, are localized in the stratum corneum where they make up 50% of the total lipid mass constituting the lipid lamellae 3. Differentiating keratinocytes synthesize ceramides with different chain lengths and hydroxylation patterns on the intracellular membranes of the secretory pathway. The first step of *de novo* biosynthesis of ceramides, a condensation reaction between l‐serine and palmitoyl‐CoA, is catalyzed by serine palmitoyltransferase (SPT). Therefore, SPT is considered a key enzyme in the regulation of ceramide production and it affects the epidermal barrier function. The major identified SPT subunits, SPTLC1, SPTLC2, and SPTLC3, are differentially expressed in human tissues 4. Farrell *et al*. 5 demonstrated that changes in *SPT* mRNA levels were reflected by comparable alterations in SPT activity. Holleran *et al*. 1 and Yang *et al*. 6 demonstrated that the application of a SPT inhibitor resulted in the delayed barrier recovery of mouse skin disrupted by tape‐stripping and acetone treatment.

Mammalian target of rapamycin (mTOR) is a serine/threonine kinase that is characteristically found in distinct multi‐protein complexes. mTOR complex (mTORC)1 contains unique accessory proteins such as Raptor, while mTORC2 contains a protein known as Rictor. The signaling of mTORC1 and mTORC2 is sensitive to inhibition by the drug rapamycin 7. Sarbassov *et al*. 8 reported that mTORC1 is much more sensitive to rapamycin than mTORC2. The downstream effects of mTORC1 are mediated by phosphorylation of eukaryotic initiation factor 4E binding protein 1 and p70 S6 kinase (S6K). mTOR is a principal effector of nutrient action, and mTOR signaling regulates lipid synthesis through various processes involving gene expression or activation 9. Moore *et al*. 10 showed that mTOR was activated in the epidermis of rats fed a HF diet. Aronova *et al*. 11 reported that the TOR signaling induces *de novo* ceramide synthesis in yeast. However, the effects of mTOR signaling on ceramide synthesis in the epidermis following the consumption of a HF diet are not well understood.

Osman *et al*. 12 reported that the treatment of rat mesangial cells with rapamycin activated transforming growth factor (TGF)‐β1/Smad signaling, which mediates a wide variety of biological functions, such as cell proliferation, differentiation, and extracellular matrix production in the skin 13. It is now clear that TGF‐β1 induced diverse cellular responses by binding to and activating specific cell‐surface receptors that have intrinsic serine/threonine kinase activity. The activated TGF‐β receptors stimulate the phosphorylation of receptor‐regulated Smad2 and Smad3 proteins. This complex translocates from cytoplasm into nucleus, where the Smads regulate the transcription of target genes. Gorshkova *et al*. 14 showed that TGF‐β1 increased the level of SPT expression in normal human lung fibroblasts. Thus, we have focused on the mTOR/TGF‐β1 pathway to regulate ceramide synthesis in the skin following the consumption of HF diets.

The aim of this study was to examine the relationship between ceramide synthesis and mTOR signaling using the skin of rats and human keratinocytes. We measured ceramide levels in rat skin by thin‐layer chromatography (TLC), the gene expression levels of *SPT* and *TGF‐β1* by using quantitative PCR and the phosphorylation status of p70 S6K, Smad2, or Smad3 by using immunoblot analysis.

## Materials and methods

### Materials

Ceramides derived from bovine brain was obtained from Doosan Serdary Research Laboratories (Eaglewood Cliffs, NJ, USA). The TaqMan universal PCR master mix core reagent kit and TaqMan Gene Expression Assays kits were obtained from Applied Biosystems (Foster City, CA, USA). HuMedia‐KG2 was purchased from Kurabo Co., Ltd. (Osaka, Japan). Recombinant human TGF‐β1 was purchased from Peprotech (Rocky Hill, NJ, USA). SB525334 was obtained from Wako Pure Chemical Industries (Osaka, Japan). Protease inhibitor cocktail were obtained from Sigma‐Aldrich Corp. (St. Louis, MO, USA). PhosSTOP phosphatase inhibitor cocktail tablets were purchased from Roche Diagnostics Corp. (Indianapolis, IN, USA). RC DC protein assay was purchased from Bio‐Rad Laboratories Inc. (Hercules, CA, USA). The Amersham Hybond‐P PVDF membrane and Amersham^™^ ECL^™^ Select Western Blotting Detection Reagents were obtained from GE Healthcare UK Ltd. (Little Chalfont, UK). ECL^™^ anti‐rabbit IgG‐horseradish peroxidase‐linked whole antibody was obtained from Rockland Immunochemicals, Inc. (Gilbertsville, PA, USA). DynaMarker Protein MultiColor III was purchased from BioDynamics Laboratory Inc. (Tokyo, Japan). Can Get Signal^®^ and PVDF Blocking Reagent for Can Get Signal^®^ were purchased from TOYOBO Co., Ltd. (Osaka, Japan). Phospho‐p70 S6K (Thr389) antibody, p70 S6K antibody, Phospho‐Smad2 (Ser465/467) polyclonal antibody, Phospho‐Smad3 (Ser423/425) polyclonal antibody and Smad2/3 antibody were obtained from Cell Signaling Technology, Inc. (Beverly, MA, USA).

### Animals and diets

Four‐week‐old male Sprague–Dawley rats (CLEA Japan, Tokyo, Japan) were individually housed in stainless‐steel cages and kept in an animal room at 23–25 °C and 50–56% humidity with a 12‐h light cycle (lights on 8:00–20:00). Rats had free access to food and drinking water. After the animals were acclimated to a normal diet based on AIN‐76 feed composition 15 for 1 week, they were divided into the three following groups: control [CO; 5% (w/w) corn oil], HF [25% (w/w) lard], and RAPA [25% (w/w) lard + 0.003% (w/w) rapamycin; LC Laboratories, Woburn, MA, USA] with 12 individuals each and were fed for 28 days. On the final day of the experimental period, the animals were dissected under sodium pentobarbital anaesthesia (body weight, 5.8 mg per 100 g).

To measure the ceramide levels, stratum corneum specimens were obtained using a stripping procedure. The remaining skin was rapidly frozen in liquid nitrogen and used for protein and mRNA quantification. This study was carried out in strict accordance with the recommendations in the Guide for the Care and Use of Laboratory Animals of the Tokyo University of Agriculture. The protocol was approved by the Committee on the Ethics of Animal Experiments of the Tokyo University of Agriculture (Permit Number: 120 001). All surgery was performed under sodium pentobarbital anesthesia, and all efforts were made to minimize suffering.

### Cell culture and treatment

The human keratinocyte cell line NHEK was obtained from Kurabo Industries, Ltd. (Osaka, Japan), and cultured in HuMedia‐KG2 containing human epidermal growth factor (0.1 ng·mL^−1^), hydrocortisone (0.5 μg·mL^−1^), gentamicin (50 μg·mL^−1^), amphotericin B (50 ng·mL^−1^) and 0.4% (v/v) bovine pituitary extract in a humidified atmosphere of 5% CO_2_ at 37 °C. Human keratinocytes were seeded in 96‐well plates in culture medium at a density of 1 × 10^4^ cells per well and were incubated for 24 h. Cells were treated with rapamycin (30 or 100 nm; Calbiochem, Darmstadt, Germany) and 0.002% dimethyl sulfoxide (DMSO) as vehicle control for 24 h. In an additional experiment, cells were seeded in 6 or 96‐well plates. Twenty‐four hours later, the cells were treated with TGF‐β1 (0 or 1.2 pm) for 0, 12, 24, 36, or 48 h. For experiments using an inhibitor, cells were treated with TGF‐β1 (0 or 1.2 pm) in the presence of varying concentrations of solubilized SB525334 (0, 1, or 3 μm) for 48 h and rapamycin (0 or 100 nm) in the presence of varying concentrations of an anti‐TGF‐β1 antibody (0, 1, or 3 ng·mL^−1^) or SB525334 (0, 1, or 3 μm) for 24 h. Both rapamycin and SB525334 were dissolved in DMSO and diluted with the medium to appropriate concentrations.

### RNA extraction and quantitative PCR

Total RNA was extracted from skin using an SV Total RNA Isolation System (Promega, Madison, WI, USA). Total RNA (2 μg) was reverse transcribed using a High Capacity cDNA Reverse Transcription Kit (Applied Biosystems) according to the manufacturer's instructions. For *in vitro* assays, the samples were prepared as described previously by Yamane *et al*. 16. Total RNA was extracted and cDNA was prepared using a TaqMan^®^ Gene Expression Cells‐to‐CT^™^ Kit (Applied Biosystems) according to the manufacturer's instructions. The amplified products were detected using the TaqMan universal PCR master mix core reagent kit and TaqMan Gene Expression Assays kit. The mRNA levels were measured using quantitative PCR with an ABI Prism 7300 apparatus (Applied Biosystems). The levels were expressed as values relative to those of *rat β‐actin* or *human GAPDH*. The primer sequences were as follows: *rat SPT* forward, 5′‐CAGTGCAGCCTGCTTTGCTA‐3′; *rat SPT* reverse, 5′‐GCCTTTCGAGGATTCTTTTGATC‐3′; and *rat SPT* probe, FAM‐CCAGAAAGGACTACAGGCATCACGCAG‐TAMRA (GenBank accession no.: XM341495); *rat β‐actin* forward, 5′‐CCCTGGCTCCTAGCACCAT‐3′; *rat β‐actin* reverse, 5′‐GATAGAGCCACCAATCCACACA‐3′ and *rat β‐actin* probe, VIC‐AGATCATTGCTCCTCCTGAGCGCAAGT‐TAMRA (GenBank accession no.: V01217); *human SPT* forward, 5′‐GGAACATCGGTGTCGTTGTG‐3′; *human SPT* reverse, 5′‐CTGCAATAGGTCCCCAACTTCA‐3′ and *human SPT* probe, FAM‐TCCTGCCACCCCAATTATTGAGTCCAG‐TAMRA (GenBank accession no.: NM_004863); *human TGF‐β1* forward, 5′‐GCGTGCTAATGGTGGAAACC‐3′; *human TGF‐β1* reverse, 5′‐CGGAGCTCTGATGTGTTGAAGA‐3′ and *human TGF‐β1* probe, FAM‐CGGAGCTCTGATGTGTTGAAGA‐TAMRA (GenBank accession no.: NM_000660). Amplification reactions were performed under the following conditions: 2 min at 50 °C and 10 min at 95 °C, followed by 50 cycles of 15 s at 95 °C and 1 min at 60 °C.

### Relative comparison of ceramides

To obtain stratum corneum lipids, a glass slide coated with glue were applied to site on the dorsolateral skin per rat and then peeled off. Total lipids in stratum corneum specimens were extracted using hexane–ethanol [19 : 1 (v/v)] as described by Thielitz *et al*. 17, filtered, evaporated to dryness at room temperature under a nitrogen stream, and finally reconstituted in chloroform to a concentration of 100 mg·mL^−1^. The lipid samples (5 μL) were spotted onto TLC plate and separated using chloroform/methanol/acetic acid [192 : 7 : 1 (v/v/v)] as the developing solvent. Total lipids in human keratinocytes were extracted using chloroform–methanol [1 : 1 (v/v)] as described by Folch *et al*. 18, evaporated to dryness at room temperature under a nitrogen stream, and finally reconstituted in chloroform–methanol [2 : 1 (v/v)]. The lipid samples (10 μL) were spotted onto TLC plate and separated using chloroform/methanol/acetic acid [190 : 9 : 1 (v/v/v)] as the developing solvent. These plates were dried, sprayed with 16% (w/v) cupric sulfate pentahydrate in 8% (w/v) phosphoric acid, and heated at 150 °C for 10 min, to visualize the target ceramide and standard bands. Ceramides derived from bovine brain were used as standard bands. The ceramide level of each test group was quantified using LAS‐3000 (Fujifilm, Tokyo, Japan) and expressed as a relative value to those of the control.

### Immunoblot analysis

Tissue lysates were extracted from the epidermis prepared using radio immunoprecipitation assay (RIPA) buffer supplemented with a protease inhibitor cocktail and a phosphatase inhibitor cocktail. Whole cell lysates were extracted from keratinocytes were prepared using the RIPA buffer. The total amount of protein in the lysates was measured with an RC DC protein assay reagent. Equal amounts of protein in the lysates were loaded and electrophoresed on 8 or 10% (w/v) SDS polyacrylamide gels and tris‐glycine electrophoresis buffer according to the standard protocol. Following the electrophoresis, the proteins were transferred onto a PVDF membrane using a transfer buffer. The membrane was subsequently blocked with PVDF Blocking Reagent for Can Get Signal^®^ for 1 h. The membrane was incubated with phospho‐p70 S6K (Thr389) antibody, phospho‐Smad2 (Ser465/467) polyclonal antibody, or phospho‐Smad3 (Ser423/425) polyclonal antibody at 4 °C overnight, washed five times with tris buffered saline containing 0.5% (v/v) Tween 20, and incubated with anti‐rabbit IgG‐horseradish peroxidase (1 : 1000 dilution in Can Get Signal^®^) for 1 h. After washing the membrane five times with tris buffered saline containing 0.5% (v/v) Tween 20, the immunoreactive bands were visualized using Amersham^™^ ECL^™^ Select Western Blotting Detection Reagents. To confirm the phosphorylation status of p70 S6K, Smad2, or Smad3, reprobing was performed with 1 : 1000 of p70 S6K or Smad2/3 antibody. The phosphorylation status of p70 S6K and Smad3 was quantified using LAS‐3000 (Fujifilm).

### Statistical analysis

Results are expressed as mean ± SE. For the *in vitro* assay, results are expressed as mean ± SD. Statistical significant differences between the two groups were determined using the Student's *t*‐test. The means of multiple groups were compared using the Student–Newman–Keuls test. *P < 0.05 and **P < 0.01 indicate values that are significantly different from the vehicle. #*P* < 0.01 indicates values that are significantly different from those of cells treated with rapamycin or TGF‐β1 alone.

## Results

### Effects of rapamycin on the phosphorylation status of p70 S6K, which indicates mTOR activation, and the levels of ceramide and the mRNA levels of *SPT* and *TGF‐β1* in the skin of rats fed HF diets

**Figure 1:**
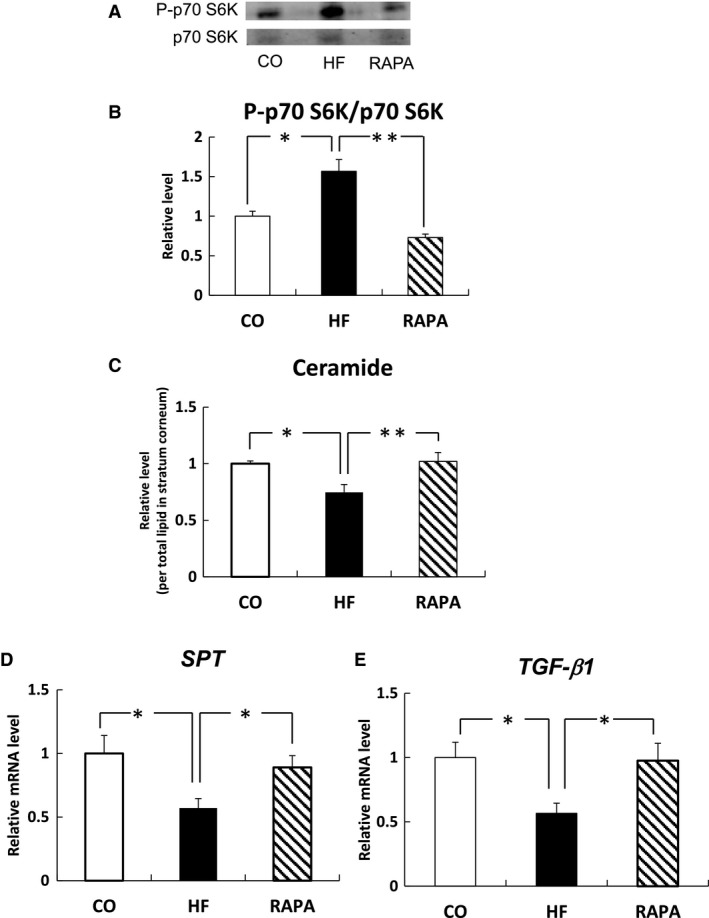
Effects of rapamycin on the phosphorylation status of p70 S6K and the levels of ceramide and the mRNA levels of *SPT* and *TGF‐β1* in the skin of rats fed HF diets. The phosphorylations of p70 S6K (Thr389) (A, B) was determined by immunoblot analysis. Bars are expressed as mean ± SE (*n* = 3). *P < 0.05 and **P < 0.01 indicate values that are significantly different from the CO group. Ceramide levels in rat skin were quantified by TLC. The ceramide levels (C) in the HF and RAPA diet groups are expressed as a fold change relative to the band intensity in the CO group. The mRNA levels of *SPT* (D) and *TGF‐β1* (E) in rat skin were measured by quantitative PCR and are expressed as a relative value to that of β‐actin. Bars are expressed as mean ± SE (*n* = 7, 12). *P < 0.05 and **P < 0.01 indicate values that are significantly different from the CO group.

We measured the phosphorylation status of p70 S6K. The phosphorylation of p70 S6K was induced in the skin of rats fed a HF diet, but this abnormality was reversed by supplementation with rapamycin (Fig. 1A,B). Figure 1C–E show the levels of ceramide and the mRNA levels of *SPT* and *TGF‐β1* in the skin of rats fed HF diets. The levels of ceramide and the mRNA levels of *SPT* and *TGF‐β1* were decreased in the skin of rats fed a HF diet, but this abnormality was reversed by supplementation with rapamycin, a pharmacological inhibitor of mTOR.

### Rapamycin induced *SPT* and *TGF‐β1* mRNA expression and TGF‐β1 induced ceramide synthesis and the mRNA levels of *SPT* in human keratinocytes

**Figure 2:**
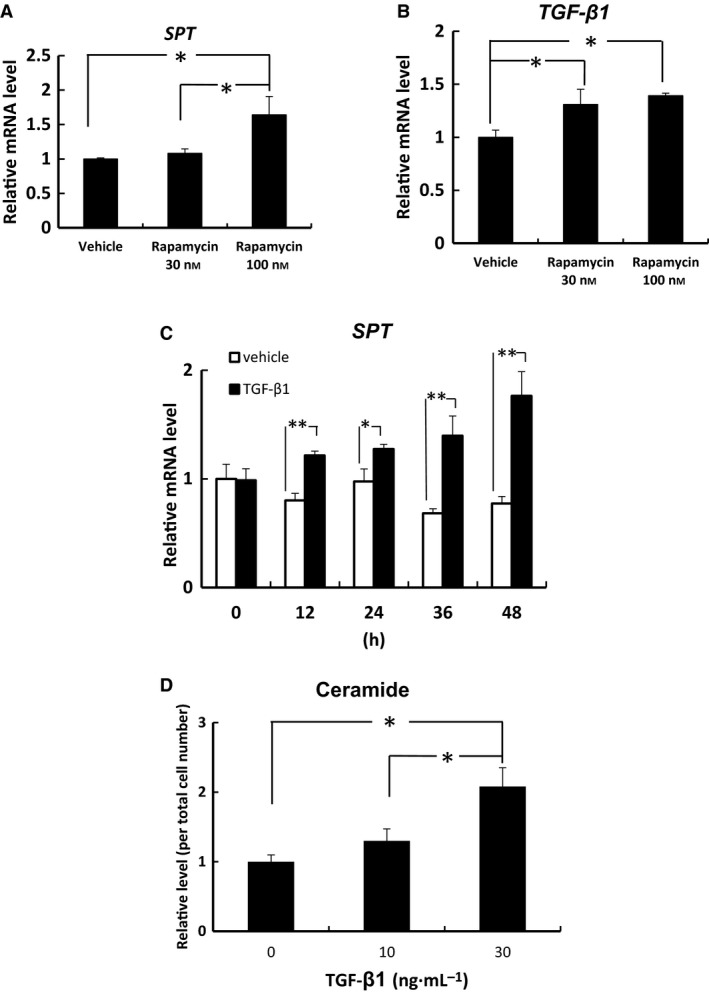
Rapamycin induced *SPT* and *TGF‐β1* mRNA expression and TGF‐β1 induced ceramide synthesis and the mRNA levels of *SPT* in human keratinocytes. The mRNA levels of *SPT* (A, C) and *TGF‐β1* (B) in human keratinocytes were measured by quantitative PCR and are expressed as a relative value to that of GAPDH. Ceramide levels in human keratinocytes were quantified by TLC. The ceramide levels (D) are expressed as a fold change relative to the band intensity in cells treated without TGF‐β1. Bars are given as mean ± SD (*n* = 3). *P < 0.05 and **P < 0.01 indicate values that are significantly different from the vehicle.

Rapamycin (100 nm) treatment significantly increased *SPT* mRNA expression in human keratinocytes (Fig. 2A). Rapamycin (30 and 100 nm) treatment significantly increased *TGF‐β1* mRNA expression in human keratinocytes (Fig. 2B). TGF‐β1 (1.2 pm) treatment significantly increased ceramide synthesis and *SPT* mRNA expression in human keratinocytes (Fig. 2C,D).

### Effects of rapamycin on TGF‐β1/Smad signaling‐induced *SPT* mRNA expression in human keratinocytes

**Figure 3:**
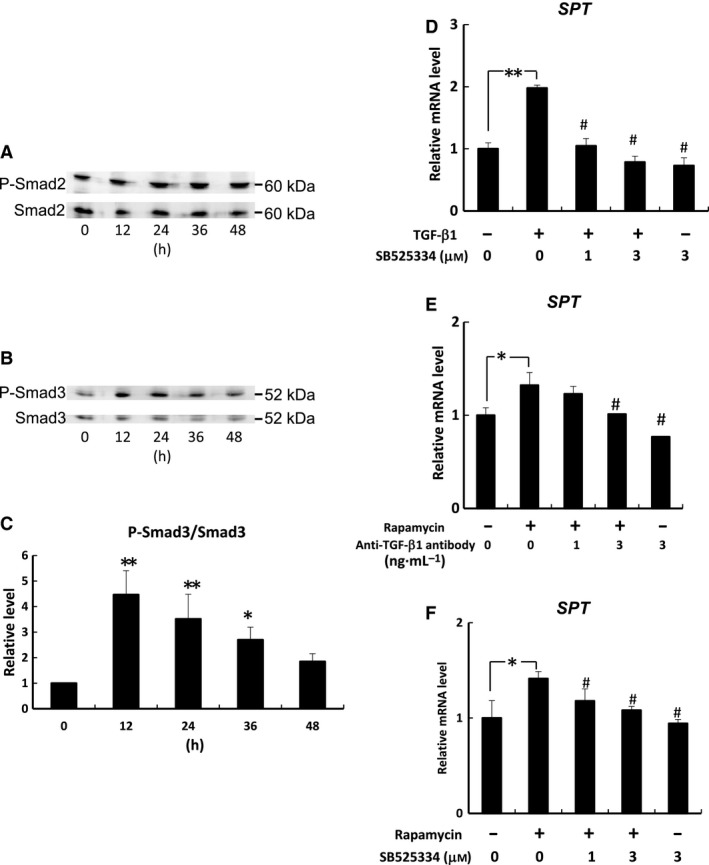
Effects of rapamycin on TGF‐β1/Smad signaling‐induced *SPT* mRNA expression in human keratinocytes. Cells were treated with TGF‐β1 (30 ng·mL^−1^) for 0, 12, 24, 36, or 48 h. The phosphorylations of Smad2 (Ser465/467) (A) and Smad3 (Ser423/425) (B, C) were determined by immunoblot analysis. *P < 0.05 and **P < 0.01 indicate values that are significantly different from 0 h. The photographs of immunoblots shown are representative of three independent experiments. *SPT* mRNA expression (D–F) in human keratinocytes was measured by quantitative PCR and is expressed as a relative value to that of *GAPDH*. Bars are expressed as mean ± SD (*n* = 3). *P < 0.05 and **P < 0.01 indicate values that are significantly different from the vehicle. ^#^ *P* < 0.01 indicates values that are significantly different from those of cells treated with rapamycin or TGF‐β1 alone.

To investigate the effects of TGF‐β1 on Smad2 and Smad3 activity, the phosphorylation status of Smad2 and Smad3 in human keratinocytes was determined after treatment with TGF‐β1 (1.2 pm) for 0, 12, 24, 36, or 48 h. The phosphorylation status of Smad2 (Ser465/467) did not change after TGF‐β1 treatment (Fig. 3A). On the other hand, TGF‐β1 treatment induced the phosphorylation of Smad3 (Ser423/425) at 12, 24, and 36 h (Fig. 3B,C). As shown in Fig. 3C, TGF‐β1‐induced *SPT* mRNA expression was blocked by SB525334, an inhibitor of TGF‐β1‐induced Smad2/3 nuclear localization. SB525334 alone had no effect on the basal *SPT* mRNA level.

As shown in Fig. 3D,E, the rapamycin‐induced *SPT* mRNA level was blocked by an anti‐TGF‐β1 antibody or SB525334 in a concentration‐dependent manner. Anti‐TGF‐β1 antibody or SB525334 alone had no effect on the basal *SPT* mRNA level.

## Discussion

Changes in mTOR activity causes skin‐related diseases, such as keloid scar, wound healing, and atopic dermatitis and alterations in ceramide content are associated with a number of skin diseases 19, 20, 21, 22. However, some aspects of the relation between mTOR and ceramide synthesis are unclear. Therefore, in this study, we examined ceramide synthesis related to the mTOR/TGF‐β1 pathway in the skin.

mTOR is a principal effector of nutrient action and forms two independent complex, mTORC1 and mTORC2. Aronova *et al*. 11 showed that the TOR signaling upregulates *de novo* ceramide synthesis in yeast. Increased ceramide synthesis is associated with the activation of SPT, the rate‐limiting enzyme in *de novo* ceramide synthesis 23. In this study, the phosphorylation of p70 S6K was induced in the skin of rats fed a HF diet, but this abnormality was reversed by supplementation with rapamycin. We also observed that the levels of ceramide and the mRNA levels of *SPT* and *TGF‐β1* were decreased in the skin of rats fed a HF diet, but this abnormality was reversed by supplementation with rapamycin, a pharmacological inhibitor of mTOR. In atopic dermatitis (AD), there was a marked reduction in the amount of ceramides in the lesional forearm skin compared with those of healthy individuals 21. In addition, increased mTOR phosphorylation in the lesional skin was observed and topical application of rapamycin remarkably improved AD symptoms in skin lesions of murine atopic dermatitis models 22. The effects of rapamycin may involve mechanisms other than mTOR in the regulation of the immune and vascular systems in AD. Therefore, we firstly propose a possibility that mTOR signaling may reduce ceramide synthesis in the skin. It is likely that rapamycin will help to improve the epidermal permeability barrier function following the consumption of a HF diet.

To clarify the mechanisms for rapamycin‐induced *SPT* mRNA expression, we examined the relationship between ceramide synthesis and the mTOR/TGF‐β1 pathway using human keratinocytes. Osman *et al*. 12 reported that rapamycin induces activity of TGF‐β1 in renal mesangial cells. In our study, rapamycin (100 nm) upregulated the gene expressions of *SPT* and *TGF‐β1* in human keratinocytes. Furthermore, an anti‐TGF‐β1 antibody almost completely inhibited rapamycin‐induced *SPT* mRNA expression in human keratinocytes. Our results show that rapamycin upregulates the gene expression of *TGF‐β1*, and these effects contribute to increases in *SPT* mRNA expression in human keratinocytes. Although low levels of rapamycin (30 nm) upregulate the gene expression of *TGF‐β1*, this concentration of rapamycin had no effect on *SPT* mRNA expression in keratinocytes. Thus, low levels of rapamycin may have no effect on activity of TGF‐β1 in keratinocytes. Taken together, our results suggest that the mTOR/TGF‐β1 pathway regulates the transcription of *SPT* in human keratinocytes.

**Figure 4:**
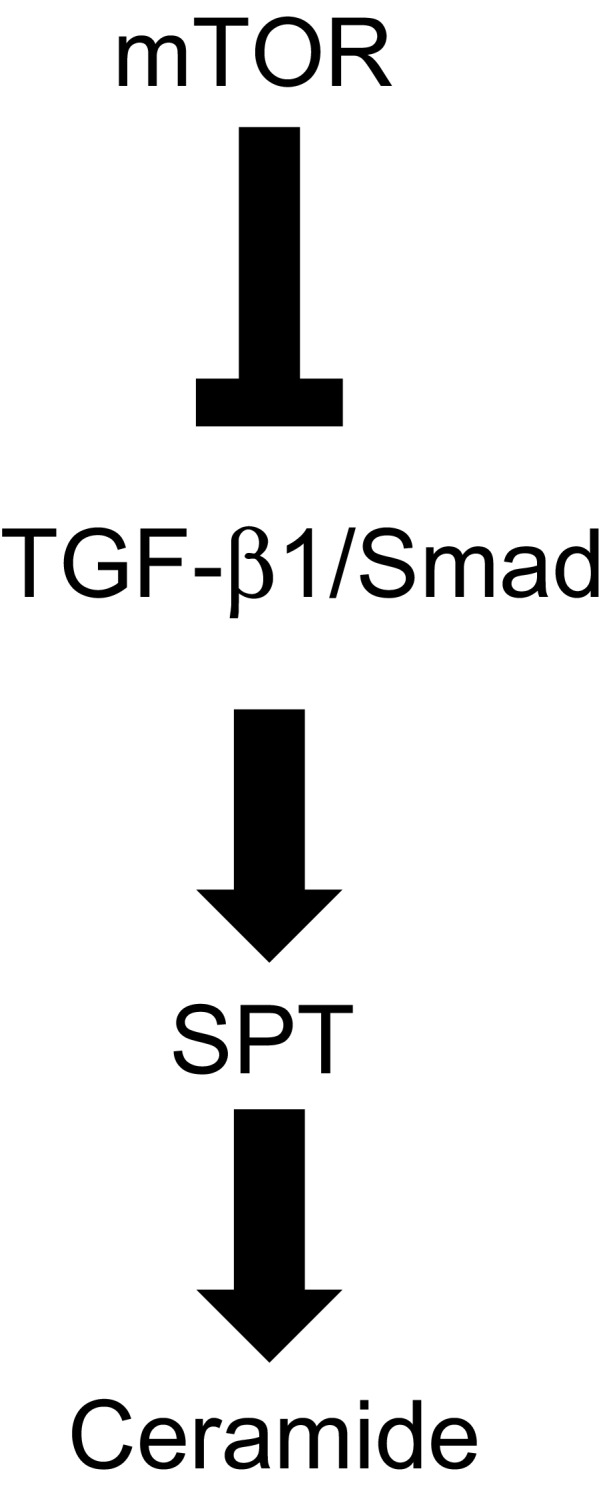
Proposed model of the mTOR/TGF‐β1 pathway in the regulation of ceramide synthesis. mTOR suppresses TGF‐β1/Smad signaling, which in turn affects ceramide synthesis.

TGF‐β1 mediates a wide variety of biological functions by binding to its high affinity type II receptor to form a tetrameric complex with type I receptor 24. The action of TGF‐β1 is mainly mediated by the Smad family of proteins and mitogen‐activated protein kinase 25. Activated TGF‐β1 receptors phosphorylate Smad proteins, which form heteromeric complexes 26. Smad2/3 complexes translocate into the cell nucleus and affect the transcriptional status of target genes in cooperation with DNA binding cofactors. Sauer *et al*. 27 showed that TGF‐β1 induced the phosphorylation of Smad3 in keratinocytes. In this study, TGF‐β1 increased the amount of ceramides in a dose‐dependent manner in human keratinocytes. We also observed that TGF‐β1 promoted Smad3 phosphorylation and *SPT* mRNA expression in human keratinocytes. In contrast, no changes in Smad2 phosphorylation were observed. Yakymovych *et al*. 27 showed that protein kinase C, a major component in the signaling pathways downstream of TGF‐β1 receptors, phosphorylated Smad3 but not Smad2. These data suggest that the action of protein kinase C is involved in TGF‐β1‐promoted Smad3 phosphorylation in human keratinocytes. Furthermore, our results showed that SB525334, an inhibitor of TGF‐β1‐induced Smad2/3 nuclear localization, almost completely inhibited TGF‐β1 or rapamycin‐induced *SPT* mRNA expression in human keratinocytes. Taken together, these results indicate that the TGF‐β1/Smad signaling pathway is modulated by the action of mTOR and involved in promoting ceramide synthesis in human keratinocytes (Fig. 4). Laplante *et al*. 28 found that mTOR impairs peroxisome proliferator‐activated receptor (PPAR)α activity by promoting the nuclear accumulation of nuclear receptor corepressor 1, a negative regulator of several nuclear receptors. We observed by quantitative RT‐PCR analysis that PPARα agonist increased *TGF‐ß1* mRNA expression in dermal fibroblasts (data not shown). Thus, it is likely that rapamycin has effects on gene expression of *TGF‐β1* by modulating PPARα activity in keratinocytes. The detailed mechanism for the up‐regulation of *SPT* gene expression by TGF‐β1/Smad signaling will be clarified by future studies, including the promoter analyses of *SPT*.

In summary, we have shown that mTOR reduces ceramide synthesis through the action of the TGF‐β1/Smad pathway in the epidermis. This is the first study to show that TGF‐β1/Smad signaling promotes *SPT* mRNA expression in human keratinocytes. In contrast, Chen *et al*. 29 showed that TGF‐β1 suppressed ceramide synthesis in NIH3T3 fibroblasts. Thus, it is likely that the control of ceramide synthesis in keratinocytes differs from that in fibroblasts. We believe that this study has shed new light on the mechanisms underlying mTOR‐induced changes in skin function.

## Author contributions

TY and YO conceived and designed the project, TY, AM, SY, SM, EC, and YT acquired the data, KI and KK analyzed and interpreted the data, TY wrote the manuscript.
